# Exploring the Impact of the Rational Antibiotic Use System on Hospital Performance: The Direct Effect and the Spillover Effect

**DOI:** 10.3390/ijerph16183463

**Published:** 2019-09-18

**Authors:** Shanshan Guo, Wenchao Du, Shuqing Chen, Xitong Guo, Xiaofeng Ju

**Affiliations:** School of Management, Harbin Institute of Technology, Harbin 150000, China; hit_guoshanshan@163.com (S.G.); 17B910027@stu.hit.edu.cn (W.D.); chenshuqinghit@gmail.com (S.C.); juxf@hit.edu.cn (X.J.)

**Keywords:** rational antibiotic use system, e-health services, hospital performance, direct effect, spillover effect

## Abstract

Irrational antibiotic usage not only causes an increase in antibiotic-borne diseases, but also inflicts pain on patients, as a result of inappropriate treatment. In order to resolve the hazards caused by irrational antibiotic usage, a kind of e-health service, the Rational Antibiotic Use System (RAUS), has been incorporated into the hospital information system. The RAUS provides doctors and patients with the functions of antibiotic usage monitoring, antibiotic information consultation and antibiotic prescription support. Though existing literature has already proved the usefulness of the RAUS on monitoring doctors’ behavior, the effects on hospital performance from an organizational perspective has rarely been measured by empirical data. Therefore, our study has explored the effects of the RAUS on the performance of a large Chinese hospital, which has implemented the RAUS since March 2014. Through empirical research, we quantified the effects of the implementation of the RAUS on a hospital’s performance from both the direct effects on the “drug income” and the spillover effect on the “treatment income”. The results indicate a significant positive spillover effect on the treatment incomes of a hospital in its inpatient activities (seen as significant in the long term) and in its outpatient activities (seen as significant in both the short and long terms). In addition, this research provides certain theoretical and practical implications for the dilemma of e-health services application in irrational antibiotic usage.

## 1. Introduction

Irrational usage of antibiotics, such as antibiotic overdose or misuse, does not only lead to excessive wastage of scarce healthcare resources, but also may cause medical accidents and thus lead to an increase in mortality rates [1,2]. To improve the rationality of antibiotic usage in prescriptions and dispensing of antibiotics, the World Health Organization (WHO) established the International Network for Rational Use of Drugs (INRUD) through the design, evaluation and promotion of effective strategies in 1989 [3]. In addition, many countries have implemented a policy of rational antibiotic usage to standardize prescribing behavior and enhance the integrity of medical units [4,5,6,7,8,9,10]. However, the dilemma of irrational antibiotic usage remains unresolved in the healthcare industry worldwide, especially in developing countries [11,12,13]. For instance, according to WHO statistics the usage rate of antibiotics in developed countries (e.g., the United States and the United Kingdom) is 22% to 25%, while it has been more than three times this amount (67% to 82%) in the past five years in China. 

Previous studies have shown that the main reason why irrational antibiotic usage is difficult to avoid is that doctors’ prescribing behavior is difficult to regulate [14]. To solve this problem, many measures have been proposed for intervening in doctors’ prescribing behaviors, such as a list of essential antibiotics/programs, standard treatment guidelines, committees comprising hospital pharmaceutical and treatment committees, rational antibiotic use training for medical staff, training for antibiotic salespersons and so on [15]. The rapid increase in e-health service has resulted in the implementation of the rational antibiotic use system (RAUS) into China’s hospitals. There are many forms of RAUSs with different functions, such as the Prescription Antibiotic Monitoring Program, the Pharmacy Management System, the Prescription Automatic Screening System and the Computerized Clinical Decision Support [16,17,18,19,20,21,22]. The functions can be summarized into: prescribing behavior monitoring, antibiotic information consultation and medication prescription support. Existing studies have shown the positive effects of RAUS usage on the individual behavior of doctors, e.g., reducing the usage of antibiotics, improving the quality of prescriptions, and so on [23,24,25,26,27].

Although existing studies have stressed the significant effects of the RAUS on doctors’ prescribing behaviors from the individual perspective [28,29], there is a lack of knowledge about how the RAUS affects hospitals’ performance from an organizational perspective. Hospitals not only provide public welfare services, but also have to endure performance pressures, as they are normal economical organizations. Therefore, the impact of the implementation of the RAUS on hospitals’ performance (such as their incomes and revenues) is one of the important determinants of a hospital to use or continually use the RAUS. As a positive effect, the RAUS may improve the efficiency of a hospital [30,31], thereby showing an increase in that hospital’s visits and performance. On the other hand, the use of the RAUS may cause an increase in the workloads of doctors and, thus, result in an increase in their human costs [32]. At the same time, the development and implementation of the RAUS will also increase the operating costs of hospitals [33,34]. Moreover, the reduction in antibiotic abuse due to a more rational use of antibiotics may also lead to a reduction in antibiotic revenue. Therefore, the impact of the RAUS on a hospital’s performance needs to be quantified. In order to fill this gap, our study will explore the impact of the RAUS on hospital performance through empirical research from the perspective of hospital organization.

As a subsystem of the HIS focusing on antibiotic usage problems, the implementation of the RAUS may result in a direct effect on the drug income of a hospital’s performance. For instance, limiting the usage of antibiotics and controlling the excessive usage of antibiotics will lead directly to a decrease in drug income. On the other hand, the effects of the RAUS may also spill over into other activities in hospitals, such as treatment activities. The spillover effect stresses that when an organization carries out an activity, it will not only produce the direct effects expected of the activity, but also produce other effects besides the those that are expected [35,36]. In summary, an activity produces external performance results beyond its implementation boundary. Hospitals that implement the RAUS, while standardizing their antibiotic usage can also improve their quality of medical treatment and the efficiency of their diagnoses and treatments, thus possibly increasing their credibility and attracting more patients. Therefore, the RAUS does generate external performance effects, that is, the spillover effect. Hence, our study focuses on a large first-level hospital in China (which we name as Hospital ABC) for empirical research, and explores the impacts of the implementation of the RAUS on hospital performance from the perspectives of the direct effect and the spillover effect, that is, the direct effect of “drug income” and the spillover effect of the “treatment income”. Therefore, through empirical research, our study explores the following questions:

(Q1) Can the implementation of the RAUS improve the performance of a hospital?

(Q2) What are the direct and spillover effects of the RAUS on hospital performance?

### 1.1. Literature Review

In order to promote the rational usage of medication, there are various forms of RAUSs with different functions in different hospitals to intervene in doctors’ prescribing behaviors. Studies on these RAUSs have verified the positive effects of the RAUS on doctors’ behaviors. For instance, in the United States, the Prescription Antibiotic Monitoring Program is widely used to address the diversion of prescription antibiotics and the abuse of antibiotic, which assists doctors in identifying antibiotic-seeking behaviors and avoid adverse antibiotic interactions effectively [18]. From a clinical perspective, a study of the Pharmacy Management System in the department of general surgery in a Chinese hospital reported that this system can standardize antibiotic accuracy apparently by improving the prescribing behaviors of doctors [16]. Another study of a Chinese hospital also verified that the introduction of the Prescription Automatic Screening System could improve pharmacy administration and result in a decrease in negative pharmacy events and enhance the rate in rational prescriptions of doctors [19]. A literature review of 396 papers on the Computerized Clinical Decision Support System, which was designed to help doctors in ensuring accurate and informed antibiotic prescriptions in pediatrics also found that antibiotic prescription support could result in the number of adverse antibiotic events and improve the adherence to guidelines [17]. 

While the impact of the RAUS on doctors’ behaviors has been extensively studied in the prior literature, there is a dearth of studies that explore the impact of the role of the RAUS on hospitals’ performance. The impact of a subsystem of HIS (i.e., the RAUS) on hospitals is essential since the usage of a system is of direct concern for hospitals. For instance, launching a new hospital information subsystem will increase the operating costs of hospitals [37]. Moreover, the implementation of the HIS may also increase the workloads and the inadaptability of doctors, thus increasing a hospital’s human costs [38,39]. Therefore, hospitals need to consider whether the usage of the RAUS can provide them with benefits as well as how they can facilitate the positive use of the RAUS.

#### 1.1.1. The Direct Effects of the RAUS on Drug Income

Besides the increase in operating costs and human costs (which are general costs of the HIS), the implementation of the RAUS may have a direct effect on the drug income of hospitals. In China, doctors and antibiotic companies share the same economic benefits [40] and moreover, the threshold for purchasing and using antibiotics is very low [41]. In order to pursue personal economic interests, some doctors prefer to provide patients with fast-acting and high-cost medicines, which increases the situation of irrational antibiotic usage in China [42]. By regulating the excessive medication (especially the antibiotics) behaviors of doctors, the implementation of the RAUS will cause doctors to prescribe lower quantities of antibiotics than they did previously, and this has a direct impact on reducing the drug incomes of related hospitals. Moreover, the RAUS limits the usage of antibiotics so that the average prices of drugs would be reduced, and hence lead to a drop in total drug income. Therefore, for improved hospital performance, the RAUS has a direct negative impact on medication income.

Thus, we hypothesize that:

**Hypothesis** **1** **(H1).** The use of the RAUS has a negative impact on a hospital’s drug income.

#### 1.1.2. The Spillover Effects of the RAUS on Treatment Incomes 

In a broad sense, a spillover is an event occurring in one context which also has an effect on other related contexts [43]. In other words, the spillover effect is one which spills over from one context to other contexts, such as the beauty of the flowers in a homeowner’s garden playing a positive role on his/her neighbours. In the same way, the usage of the RAUS in a hospital may also experience the spillover effect in addition to its influence on rational drug use, and in addition, the impact of the RAUS usage may overflow positively into the income of a hospital’s treatment. 

Through the use of the RAUS, doctors’ behaviors will adhere more to a hospital’s guidelines and the occurrence of antibiotics accidents such as incompatible antibiotics, antibiotics overdose, adverse antibiotics interactions and the effective avoidance of contraindicated antibiotics dosages [44]. As fewer antibiotic accidents occur through the implementation of the RAUS, the attitudes of patients toward doctors will likewise gradually improve. In addition, the RAUS can also provide medication information to patients such as antibiotics usage guidelines, the introduction of the dangers of antibiotics overuse and antibiotic precautions, and so on. This provides a channel for patients to better understand doctors’ medication behaviors, which will also have a positive impact on the doctor-patient relationship in the long term. On the other hand, the usage of the RAUS plays a positive role in improving doctors’ medical efficiency and accuracy [16], as they can easily access medical information and check the antibiotics usage in real time without merely relying on their own memories and experiences. Therefore, the medical quality and medical efficiency of doctors will be effectively improved with the use of the RAUS, and consequently improve a hospital’s reputation. In conclusion, by gradually improving the doctor-patient relationship and medical efficiency, the effect of implementing the RAUS will spillover from antibiotics usage to hospital treatment. More patients would like to go to such a hospital and this hospital can treat more patients, hence leading to an increase in this hospital’s treatment income. As the spillover effect needs time to manifest itself, we postulate a significant spillover effect of the RAUS on a hospital’s treatment income while it is insignificant in the short term.

Thus, we hypothesize that:

**Hypothesize** **2** **(H2a).** The usage of the RAUS does not have a short-term impact on a hospital’s treatment income.

**Hypothesis** **2** **(H2b).** The usage of the RAUS has a positive spillover effect on a hospital’s treatment income in the long term.

### 1.2. Theoretical Framework

**Figure 1:**
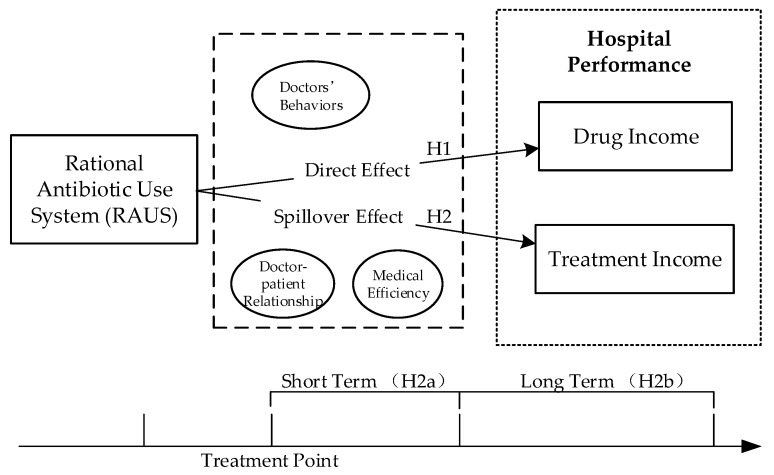
Research Model.

We thus derive a conclusion that the use of the RAUS has a negative impact on a hospital’s drug income (Hypothesis 1); the usage of the RAUS does not have a short-term impact on a hospital’s treatment income (Hypothesis 2a), but has a positive spillover effect on a hospital’s treatment income in the long term (Hypothesis 2b). Therefore, based on the literature review and hypotheses above, this study proposes an integrated research model (see Figure 1).

## 2. Methods 

### 2.1. Setting

In August 2009, a policy had been enacted to improve China’s antibiotics supply system and address the problems of antibiotics abuse [45]. In order to respond to this national policy and standardize the antibiotics usage of doctors, several Chinese hospitals attempted to introduce the RAUS to manage doctors’ behaviors regarding antibiotics usage. In March 2014, Hospital ABC with which we collaborated implemented the RAUS, thus offering a natural experiment for analyzing the effects of the RAUS. 

Hospital ABC is a large public hospital with more than 60 medical departments, and was established in 1972 in Beijing, the capital of China. This hospital is a general hospital which integrates medical treatment, teaching, scientific research, and disease prevention, and is equipped with advanced medical equipment. It has more than 60,000 outpatients visiting it annually. 

Hospital ABC initiated the RAUS in March 2014, which is installed in the workstations of doctors in each department and the rooms of the office for patients. The RAUS has structured, split and stored massive antibiotics information and abundant clinical pharmaceutical literature to form a database of rational antibiotics use with complete and accurate information. It is used to analyze whether the use of a particular antibiotic is reasonable and generates corresponding analysis results, such as the suitability and the standardization of the medical order, which can provide support for clinical information as well as provide antibiotics usage reminders for medical staff. In addition, the system also has a patient App which can be installed on the patient’s mobile phone to provide patients with antibiotics inquiry channels to help patients to increase knowledge related to medication safety, so as to enable them to better understand the rational prescribing behaviors of doctors.

After discussing the ethical problems with Hospital ABC’s administrators and public officials, we were able to gain access to its database after ensuring the protection of the privacy of information used and to conduct our analysis of the direct and indirect impacts of the RAUS on hospital performance by using the hospitalization data of Hospital ABC.

### 2.2. Data and Variables

Using the Hospital Information System (HIS), we collected data from Hospital ABC and accessed the data for inpatient and outpatient information. The panel data included the diagnostic information for each department from January 2013 to December 2015, spanning its implementation of the RAUS on March 2014, which included the number of inpatients, the number of outpatients, the departments engaged in various treatments, the dates of the treatments, and so on. Such data was used to build econometric models to enable a detailed analysis on the impacts of the RAUS on hospital performance from the direct and indirect perspectives. The hospital performance is the summation of all the departments’ performance so that the department performance can be used as an alternative measurement of the hospital performance. Benefit from the richness of the data set, we carried out our research using the panel data at the departmental levels instead of using the time series data at the hospital level to get a more detailed and credible analysis. We also compared the long-term and short-term effects of the RAUS on the hospital’s performance.

Accordingly, we constructed departmental-level variables from the HIS as follows. The dependable variables, drug income and treatment income were used to measure the performance of the hospital. In addition, drug income, the incomes of antibiotics (antibiotic) and other types of medicine (medicine), which included traditional Chinese medicine and Western medicine (but excluding antibiotics), were also included. Moreover, treatment income included both inpatient and outpatient services. The incomes of inpatient treatment (inpatient) were derived from the summary of the patient registration and medical treatment fees, while the income of outpatient treatment (outpatient) was derived from the total treatment incomes of patients besides drug incomes, such as the costs of beds, operations, medical examination, and so on. Regarding the independent variables, *afterTreatment* was used as a dummy variable to represent whether the department had implemented the RAUS. The implementation of the RAUS is regarded as a form of treatment, and following this treatment, this variable has a value of 1, otherwise it is 0. Considering that the income of each department may be affected by the number of patients in a month, we added the number of registration patients to the model as a control variable (*RegisterNum*). Table 1 presents the definitions of all the variables.

After data processing, the data included 888 hospitalization records and 3650 outpatient records at the departmental-level and the observation period was 15 months before and after the implementation of the RAUS, respectively. In order to better understand the effects of the RAUS on Hospital ABC’s income, we investigated the hypotheses with both short-term data (5 months before and after the implementation of the RAUS) and long-term data (15 months before and after the implementation of the RAUS) to check the direct effects and the spillover effects of the RAUS usage. The descriptive statistical analysis of the variables used in our model is shown in Table 2 and Table 3.

### 2.3. Research Model

In order to test our hypotheses, we formulated four models to explore the effects of the implementation of the RAUS on Hospital ABC’s performance: i.e., drug and treatment incomes. We used the income from antibiotics and other incomes from medicine to measure the hospital’s drug income; and we also used the outpatient and inpatient treatment incomes to measure the hospital’s treatment income. We also added the patient number to the model as the control variable.

The first model that explores the impact of the RAUS on antibiotic use was formulated as follows:

(1) $$\ln\left(antibiotic_{it}\right)=\beta_{0}*afterTreatment_{t}+\beta_{1}*\ln\left(patients_{it}\right)+\varepsilon_{it}$$

Then, we use the income from other medication as dependent variable to build the second model:

(2) $$\ln\left(medicine_{it}\right)=\beta_{0}+\beta_{1}*afterTreatment_{t}+\beta_{2}*\ln\left(patients_{it}\right)+\varepsilon_{it}$$

After testing the system’s impact on the drug income of Hospital ABC, we use the outpatient treatment income as the dependent variable to formulate the third model as follows:

(3) $$\ln\left(outpatient_{it}\right)=\beta_{0}+\beta_{1}*afterTreatment_{t}+\beta_{2}*\ln\left(patients_{it}\right)+\varepsilon_{it}$$

Finally, we use the inpatient treatment income as the dependent variable to derive our final model:

(4) $$\ln\left(inpatient_{it}\right)=\beta_{0}+\beta_{1}*afterTreatment_{t}+\beta_{2}*\ln\left(patients_{it}\right)+\varepsilon_{it}$$

Let i=1, 2…N be the index of each department and t=1, 2…M be the index of the month. For each model, we use different observation windows to examine the long-term and the short-term effects of the RAUS.

## 3. Results

### 3.1. Main Results

The regression results of the system’s impact on Hospital ABC’s performance (drug income and treatment income), which was derived using ordinary least squares, are presented in Table 4 and Table 5, respectively.

First, we presented the long-term and the short-term effects of the RAUS on the use of antibiotics in Column 1 and Column 2 of Table 4. Based on these two columns, we can perceive that the estimated coefficients of afterTreatment are both statistically negative (β = −0.053, *p* < 0.01; β = −0.048, *p* < 0.01). Accordingly, we can verify that the RAUS can reduce the use of antibiotics in both the short and long terms. Following this, we explored how the income of other medications changed after the implementation of the RAUS in the second model and the results are presented in Column 3 and Column 4 of Table 4. We note that coefficient of afterTreatment is not statistically significant (β = 0.026, *p* > 0.1;) in Column 3 but significant (β = 0.053, *p* < 0.1) in Column 4. The estimated results in the last two columns stress that the RAUS has a positive effect on the incomes of other forms of medication in the short term but not in the long term. 

Second, we explored how the RAUS impacts the outpatient and inpatient treatment incomes and present the regression results in Table 5. It can be observed from the first two columns of Table 5, that the coefficients of afterTreatment are both statistically positive (β = 0.165, *p* < 0.01; β = 0.110, *p* < 0.05) which indicates that the system has a positive influence on the outpatient income in both the long and short terms. Similarly, the results in the last two columns indicate the change in inpatient income after the use of the system. From estimated coefficients in these two columns, we observe that the coefficient is significant in Column 3 but not in Column 4 (β = 0.240, *p* < 0.05; β = 0.032, *p* < 0.1), which means the system can improve inpatient income in the short term but not in the long term.

### 3.2. Robustness Check

In order to check the robustness of the above results, we add the individual fixed effects to our models so that we can control the bias resulting from the variables that are only related to each department but are not related to time. The new regression results are presented in Table 6 and Table 7. Based on the coefficients of the *AfterTreatment*, we observe that the results are all consistent with the results in the previous tables, which does indicate that our regression results are stable after considering other factors that are only related to each department.

## 4. Discussions

### 4.1. Key Findings

Our study mainly explored the impacts of the RAUS on hospital performance, including the direct effects of the “drug income” and the spillover effect of the “treatment income” in hospitals. Firstly, from the results of the evaluation of our model, we observe that the implementation of the RAUS has inhibited the irrational use of antibiotics ((β_antibiotics_long_term_ = −0.053, *p* < 0.01; β_antibiotics_lshort_term_ = −0.048, *p* < 0.01); in other words, the income from antibiotics in general dropped after the RAUS was adopted. Secondly, in the short term, the income of the other forms of medication indicated an upward trend (β_medicine_short_term_ = 0.053, *p* < 0.1), which indicated that a decrease in antibiotic use would be offset by an increase in other forms of medication in the short term. To offset the intervention of the RAUS, doctors may choose to use other forms of alternative medication (e.g., proprietary Chinese medicine, Chinese herbal medicine and other forms of western medicine) in the short term, however, the substitution effect is rendered ineffective with the prolonged use of the RAUS in the long term (β_medicine_long_term_ = 0.026, *p* > 0.1). Therefore, the RAUS has succeeded in standardizing the use of antibiotics and other forms of medication in the long term. In other words, the RAUS controls the irrational use of antibiotics and other drugs, but at the same time, it also causes a reduction in drug income, thereby reducing hospital performance. Thirdly, in terms of treatment income (outpatient and inpatient), we do perceive from the results of the inpatient income, that in the short term, the implementation of the RAUS has no significant impact on inpatient income (β_inpatient_short_term_ = 0.032, *p* > 0.1), but in the long term, the inpatient income is seen to increase significantly (β_inpatient_long_term_ = 0.240, *p* < 0.05). This may be due to the use of the RAUS, which by regulating the behavior of doctors, effectively alleviates the doctor-patient relationship, and improves medical efficiency, thereby generating spillover performance. In addition, we can observe from the outpatient income results, that regardless of the duration being short or long terms, outpatient income is seen to significantly increase, and does impose a greater impact in the long term (β_outpatient_long_term_ = 0.165, *p* < 0.01; β_outpatient_short_term_ = 0.110, *p* < 0.05). This shows that the implementation of the RAUS has a faster spillover effect on the simple process of outpatient diagnosis and treatment. In summary, the spillover effect of the system for inpatients is significant in the short term but not in the long term. However, the spillover effect is significant in both the short and long terms for outpatient income. Moreover, this dilemma also indicates that the RAUS system’s spillover effect works at a faster pace for simple treatment processes (outpatient) than for complex ones (inpatient). Furthermore, we added the individual fixed effect to test the robustness of our models. Finally, in retrospect to our original data set, patient-level statistics have been made to verify our results. The statistics certify that, after the implementation of the RAUS in the hospital for one year, the efficiency indicators have been significantly improved. Moreover, the daily use of antibiotics has decreased from 300–400 patients to 60–70 patients, the proportion of antibiotics used in outpatient clinics has decreased by 2.74% and the proportion of drugs used in outpatient clinics has decreased by 6%, In addition, the number of outpatient clinics has been increased by 2.02% since March 2015. Consequently, it can be observed that the results of our model are consistent and credible.

### 4.2. Theoretical Implications

Although there has been a plethora of literature on rational antibiotics usage [46], irrational antibiotics usage is still prevalent in practice in the medical field, especially in developing countries where the abuse of antibiotics is particularly serious [47]. Therefore, more in-depth theoretical and practical exploration based on innovation e-health services is still needed on the study of rational antibiotics usage. We provide the empirical results of our study. Now, we highlight our theoretical contributions. Firstly, previous studies have verified the impact of the implementation of the RAUS from an individual perspective, i.e., it results in the standardization of doctors’ prescribing behaviors [48,49]. However, few studies have explored the direct and spillover impacts of the implementation of the RAUS at the organizational level, in terms of hospital performance. In our study, a first-level hospital in China which has implemented the RAUS was used as our research object. An empirical research method was used to explore the impact of the implementation of the RAUS on hospital performance, and the extent of the impact of the RAUS on hospital income was clearly measured from different angles. Secondly, regarding the regulating of antibiotics usage by the RAUS [47], our study has explored the direct impact of the RAUS on hospital performance, and consequently, drug income reflected a downward trend in the long term. We also found that, while improving doctors’ medication behavior by providing rational medication standards, the use of the RAUS did indeed caused a reduction in drug income directly. On the other hand, the results also indicate that the loss of drug income could be compensated by an increase in treatment income. In addition, at the organizational level, our study proposes that, through improving doctor-patient relationships and improving medical efficiency in the long term, the RAUS can attract more revenue to hospitals through the spillover effect, which also provides a theoretical basis for further exploring the mechanisms of the RAUS.

### 4.3. Practical Implications

This study also provides relevant practical implications. Firstly, our research results verify that the implementation of the RAUS shows no obvious improvement or even a downward trend in hospital performance in the short term, but in the long term, the implementation of the RAUS has a positive spillover effect on hospital performance. This proves the effectiveness and necessity of the implementation of the RAUS in hospitals. It aids the further implementation of the rational antibiotics usage policy, promotes the application of the e-health services in hospitals to standardize doctors’ prescribing behaviors, and expedites the wider implementation of the RAUS. Secondly, through the analysis of the results of the drug income, it can be seen that in the short term, drug income shows an upward trend after the implementation of the RAUS which implies the possibility of doctors using other drugs instead of antibiotics. Therefore, hospital managers are reminded that in the early stages of the implementation of the RAUS, doctors need to strictly monitor their behavior regarding the use of other drugs instead of antibiotics. Finally, according to the results on treatment income, the effect of the RAUS on inpatient income is not obvious in the short term, but significantly improves in the long term. Hospital managers are reminded to maintain confidence in the RAUS in the initial stage of the implementation of the RAUS. The results also indicate that in the short term, the implementation of the RAUS may lead to a quick spillover effect on the income from outpatient treatment (which has a simpler treatment process). Therefore, it is proposed to examine the effects of the implementation of the RAUS at an earlier stage, such as by choosing pilot departments and using simple treatment processes.

### 4.4. Limitations and Directions for Future Research

Some limitations of this study need to be considered. Firstly, although our hypotheses have been reasonably verified and our findings reflect a certain robustness, this study has only applied the systematic data of one hospital in China. We need to include more hospitals from other countries or regions to ensure the validation of our research, such as eliminating the impacts of the characteristics of regional hospitals by using the Difference in Differences method, and thus obtain more accurate causal conclusions. Secondly, rational drug usage behavior could be affected by doctor’s habits or other cognitive aspects, which may lead to a deviation in the results of our analysis. However, such data cannot be obtained from the HIS. Though our study is explored from the perspective of organization, which may weaken the doctor’s personal characteristics of deviation, hence in order to make the result more precise, we hope to add such data for further verification in future studies. 

## 5. Conclusions

In our paper, we extended the effects of the Rational Antibiotics Use System (RAUS), a kind of e-health services aim at rational antibiotics usage, on doctors’ individual behaviors in relation to the performance of hospitals at the organizational level, including the direct effects on the “drug income” and the spillover effect on the "treatment income”. Based on the empirical results, we find that the implementation of the RAUS can effectively improve a hospital’s performance in the long term. Specifically, regarding the direct effect, the results indicate that the application of the RAUS has an inhibitory effect on the irrational use of antibiotics in both the long and short terms. Alternatively, the income of other forms of medication increased in the short term, however, this dilemma can also be standardized for a long period of the implementation of the RAUS. Therefore, reaching a long-term goal for controlling the use of antibiotics and other forms of irrational antibiotics usage (e.g., antibiotics overdose), the implementation of the RAUS will also result in a reduction of a hospital’s drug income. Concerning the spillover effect, the positive effects of the RAUS on hospital performance have been significantly verified in both the inpatient and outpatient processes. Moreover, the spillover effect of the RAUS on treatment income occurs at a faster pace for outpatient than inpatient treatments, and has a greater impact in the long term. While quantifying the effects of the RAUS on hospital performance (including the direct effect and the spillover effect), the results also shed light on the practical implications of the e-health services on rational antibiotics usage. 

## Figures and Tables

**Table 1:** Description of Variables.

Variable Type	Name of Variable		Description
Dependent variable	Drug Income	antibiotic	The drug income from antibiotics
		medicine	The drug income from other medications besides antibiotics
	Treatment Income	outpatient	The treatment income of outpatients including registration, diagnosis and treatment fees
		inpatient	The treatment income of inpatients (the total inpatient income excludes drug income)
Independent variable		After Treatment	If the department has implemented the RAUS: 1; otherwise: 0
Control variable		Patients	The number of patients in a certain department during a month

**Table 2:** Statistical Description of Variables (outpatient).

Variable	Obs	Mean	Std.Dev.	Min	Max
Outpatient	3650	9750.625	53,172.68	0	730,000
afterTreatment	3650	0.599	0.49	0	1
Patients	3650	1887.515	9832.374	1	126,000

**Table 3:** Statistical Description of Variables (inpatient).

Variable	Obs	Mean	Std.Dev.	Min	Max
afterTreatment	888	0.606	0.489	0	1
Antibiotic	888	0.574	0.279	0	1
Medicine	888	350,000	325,000	0	2,950,000
Inpatient	888	71,357.47	69,136.49	0	482,000
Patients	888	84.435	99.307	1	707

**Table 4:** Parameter Estimates of the Drug Income Model.

Model Factors	Antibiotics		Other Medicine	
	(1)	(2)	(3)	(4)
	Long term	Short term	Long term	Short term
AfterTreatment	−0.053 ***	−0.048 ***	0.026	0.053 *
	(0.012)	(0.013)	(0.035)	(0.029)
Patient	−0.005	0.022	1.078 ***	1.099 ***
	(0.017)	(0.029)	(0.053)	(0.076)
Constant	0.629 ***	0.527 ***	7.863 ***	7.780 ***
	(0.074)	(0.119)	(0.309)	(0.396)
Obs.	698	241	743	247
R-squared	0.0604	0.0637	0.5881	0.5598

Standard errors are in parenthesis. *** *p* < 0.01, ** *p* < 0.05, * *p* < 0.1.

**Table 5:** Parameter Estimates of Treatment Income Model.

Model Factors	Outpatient		Inpatient	
	(1)	(2)	(3)	(4)
	Long term	Short term	Long term	Short term
AfterTreatment	0.165 ***	0.110 **	0.240 **	0.032
	(0.033)	(0.049)	(0.102)	(0.057)
Patient	1.149 ***	1.087 ***	1.034 ***	0.952 ***
	(0.044)	(0.043)	(0.137)	(0.085)
Constant	0.112	0.438 *	6.321 ***	6.756 ***
	(0.222)	(0.239)	(0.582)	(0.446)
Obs.	3089	1021	743	198
R-squared	0.7605	0.6382	0.3179	0.3025

Standard errors are in parenthesis. *** *p* < 0.01, ** *p* < 0.05, * *p* < 0.1.

**Table 6:** Robustness Check of the Drug Income Model.

Model Factors	Antibiotic		Other Medicine	
	(1)	(2)	(1)	(2)
	Long term	Short term	Long term	Short term
AfterTreatment	−0.053 ***	−0.052 ***	0.026	0.052 *
	(0.012)	(0.013)	(0.035)	(0.030)
Patient	−0.000	0.049	1.080 ***	1.111 ***
	(0.019)	(0.037)	(0.052)	(0.087)
Constant	0.611 ***	0.410 ***	8.008 ***	7.834 ***
	(0.072)	(0.146)	(0.206)	(0.346)
Obs.	698	241	743	247
R-squared	0.061	0.069	0.588	0.560

Standard errors are in parenthesis. *** *p* < 0.01, ** *p* < 0.05, * *p* < 0.1.

**Table 7:** Robustness Check of the Treatment Income Model.

Model Factors	Outpatient		Inpatient	
	(1)	(2)	(3)	(4)
	Long term	Short term	Long term	Short term
AfterTreatment	0.165 ***	0.113 **	0.242 **	0.045
	(0.034)	(0.050)	(0.102)	(0.058)
Patient	1.145 ***	1.049 ***	1.039 ***	0.897 ***
	(0.048)	(0.063)	(0.147)	(0.101)
Constant	0.171	0.735 **	6.348 ***	7.041 ***
	(0.259)	(0.341)	(0.579)	(0.391)
Obs.	3089	1021	743	198
R-squared	0.761	0.638	0.318	0.303

Standard errors are in parenthesis. *** *p* < 0.01, ** *p* < 0.05, * *p* < 0.1.

## Data Availability

The datasets generated and analyzed during the current study are not publicly available due to the data confidentiality agreement with the hospital but are available from the corresponding author on reasonable request.

## References

[1] Laing R.O. (1990). Rational drug use: An unsolved problem. Trop. Doct..

[2] Nutt D., King L.A., Saulsbury W., Blakemore C. (2007). Development of a rational scale to assess the harm of drugs of potential misuse. Lancet.

[3] World Health Organization (2002). Promoting Rational Use of Medicines: Core Components.

[4] Weekes L.M., Brooks C. (2010). Drug and Therapeutics Committees in Australia: Expected and actual performance. Brit. J. Clin. Pharmacol..

[5] Rechel B., Lessof S., Busse R., Mckee M., Figueras J., Mossialos E., Ginneken E.V. (2016). A Framework for Health System Comparisons: The Health Systems in Transition (HiT) Series of the European Observatory on Health Systems and Policies.

[6] Li X., Lu J., Hu S., Cheng K., De Maeseneer J., Meng Q., Mossialos E., Xu D.R., Yip W., Zhang H. (2017). The primary health-care system in China. Lancet.

[7] Dal Pizzol T.D.S., Fontanella A.T., Ferreira M.B.C., Bertoldi A.D., Borges R.B., Mengue S.S. (2019). Analgesic use among the Brazilian population: Results from the National Survey on Access, Use and Promotion of Rational Use of Medicines (PNAUM). PLoS ONE.

[8] Calikoglu E., Koycegiz E., Kosan Z., Aras A. (2019). Rational drug use and prescribing behavior of family physicians in Erzurum, Turkey. Niger. J. Clin. Pract..

[9] Chareonkul C., Khun V.L., Boonshuyar C. (2002). Rational drug use in Cambodia: Study of three pilot health centers in Kampong Thom Province. Southeast Asian J. Trop. Med. Public Health.

[10] Hogerzeil H.V. (1995). Promoting rational prescribing: An international perspective. Br. J. Clin. Pharmacol..

[11] Alanis A.J. (2005). Resistance to Antibiotics: Are We in the Post-Antibiotic Era?. Arch. Med. Res..

[12] Kakkar M., Walia K., Vong S., Chatterjee P., Sharma A. (2017). Antibiotic resistance and its containment in India. BMJ.

[13] Hogerzeil H.V., Ross-Degnan D., Laing R., Ofori-Adjei D., Santoso B., Chowdhury A.A., Das A., Kafle K.K., Mabadeje A., Massele A. (1993). Field tests for rational drug use in twelve developing countries. Lancet.

[14] Kunin C.M. (1993). Resistance to antimicrobial drugs—A worldwide calamity. Ann. Intern. Med..

[15] Ross-Degnan D., Laing R., Quick J., Ali H.M., Ofori-Adjei D., Salako L., Santoso B. (1992). A strategy for promoting improved pharmaceutical use: The International Network for Rational Use of Drugs. Soc. Sci. Med..

[16] Bao L., Wang Y., Shang T., Ren X., Ma R. (2013). A novel clinical pharmacy management system in improving the rational drug use in department of general surgery. Indian J. Pharm. Sci..

[17] Stultz J.S., Nahata M.C. (2012). Computerized clinical decision support for medication prescribing and utilization in pediatrics. J. Am. Med Inform. Assoc..

[18] Shipra J., Prerna U., Jaswant G., Abhijit K., Pushpawati J., Vikas S., Moghe V.V. (2015). A systematic review of prescription pattern monitoring studies and their effectiveness in promoting rational use of medicines. Perspect. Clin. Res..

[19] Wei-Bo W., Chen C.M. (2018). Practice of Prescription Automatic Screening System in Hospital Pharmacy Management. Hosp. Manag. Forum.

[20] Carling C.L., Kirkehei I., Dalsbø T.K., Paulsen E. (2013). Risks to patient safety associated with implementation of electronic applications for medication management in ambulatory care—A systematic review. BMC Med. Inform. Decis. Mak..

[21] Lyell D., Magrabi F., Raban M.Z., Pont L.G., Baysari M.T., Day R.O., Coiera E. (2017). Automation bias in electronic prescribing. BMC Med. Inform. Decis. Mak..

[22] Soleymani F., Abdollahi M. (2012). Management information system in promoting rational drug use. Int. J. Pharmacol..

[23] Ratanawijitrasin S., Soumerai S.B., Weerasuriya K. (2001). Do national medicinal drug policies and essential drug programs improve drug use? A review of experiences in developing countries. Soc. Sci. Med..

[24] Dayer L., Heldenbrand S., Anderson P., Gubbins P.O., Martin B.C. (2013). Smartphone medication adherence apps: Potential benefits to patients and providers. J. Am. Pharm. Assoc..

[25] Goyal R., Pareek P. (2013). A review article on prescription behavior of doctors, influenced by the medical representative in Rajasthan, India. IOSR J. Bus. Manag..

[26] Le Grand A., Hogerzeil H.V., Haaijer-Ruskamp F.M. (1999). Intervention research in rational use of drugs: A review. Health Policy Plann..

[27] Liu C., Liu C., Wang D., Zhang X. (2019). Knowledge, Attitudes and Intentions to Prescribe Antibiotics: A Structural Equation Modeling Study of Primary Care Institutions in Hubei, China. Int. J. Environ. Res. Public Health.

[28] Soleymani F., Ahmadizar F., Meysamie A., Abdollahi M. (2013). A survey on the factors influencing the pattern of medicine’s use: Concerns on irrational use of drugs. J. Res. Pharm. Pract..

[29] Hua X., Huang Z., Yang R., Chen C., Hu Q., Chen D., Gu J. (2013). International Conference on Web Information Systems Engineering. Semantic Approach for Rational Use of Antibiotics: A Perspective from Clinical Research.

[30] Chisholm D., Evans D.B. (2010). Improving Health System Efficiency as a Means of Moving Towards Universal Coverage.

[31] Pitaknetinan K., Tangcharoensathien V., Supachutikul A., Bennett S., Mills A. (1999). Profit, payment and pharmaceutical practices: Perspectives from hospitals in Bangkok. Health Policy.

[32] Meier D.E., Back A.L., Morrison R.S. (2001). The inner life of physicians and care of the seriously ill. JAMA.

[33] Paul S.M., Mytelka D.S., Dunwiddie C.T., Persinger C.C., Munos B.H., Lindborg S.R., Schacht A.L. (2010). How to improve R&D productivity: The pharmaceutical industry’s grand challenge. Nat. Rev. Drug Discov..

[34] Hillestad R., Bigelow J., Bower A., Girosi F., Meili R., Scoville R., Taylor R. (2005). Can electronic medical record systems transform health care? Potential health benefits, savings, and costs. Health Aff..

[35] Young Bong C., Vijay G. (2012). The Impact of IT-Related Spillovers on Long-Run Productivity: An Empirical Analysis. Inf. Syst. Res..

[36] Atasoy H., Chen P.Y., Ganju K.K. (2015). The Spillover Effects of Health IT Investments on Regional Heath Care Costs. Soc. Sci. Electron. Publ..

[37] Thouin M.F., Bardhan I. (2009). The Effect of Information Systems on the Quality and Cost of Healthcare Processes: A Longitudinal Study of US Hospitals. Arch. Neurol. Psychiatry.

[38] Askren W.B. (1973). Human Resources and Personnel Cost Data in System Design Tradeoffs: And How to Increase Design Engineer Use of Human Data.

[39] Sharma L., Chandrasekaran A., Boyer K.K., Mcdermott C.M. (2016). The impact of Health Information Technology bundles on Hospital performance: An econometric study. J. Oper. Manag..

[40] Chen M., Wang L., Chen W., Zhang L., Jiang H., Mao W. (2014). Does economic incentive matter for rational use of medicine? China’s experience from the essential medicines program. Pharmacoeconomics.

[41] Su M., Zhang Q., Bai X., Wu C., Li Y., Mossialos E., Mensah G.A., Masoudi F.A., Lu J., Li X. (2017). Availability, cost, and prescription patterns of antihypertensive medications in primary health care in China: A nationwide cross-sectional survey. Lancet.

[42] Jing W. (2004). Effect of basic drugs and rational drug use policies on rural drug use. Med. Soc..

[43] Garmaise M.J., Natividad G. (2016). Spillovers in Local Banking Markets. Rev. Corp. Financ. Stud..

[44] Eguale T., Buckeridge D.L., Verma A., Winslade N.E., Benedetti A., Hanley J.A., Tamblyn R. (2016). Association of Off-label Drug Use and Adverse Drug Events in an Adult Population. JAMA Intern. Med..

[45] Liu G.G., Vortherms S.A., Hong X. (2017). China’s health reform update. Annu. Rev. Public Health.

[46] Chao J., Gu J., Zhang H., Chen H., Wu Z. (2018). The Impact of the National Essential Medicines Policy on Rational Drug Use in Primary Care Institutions in Jiangsu Province of China. Iran. J. Public Health.

[47] Ford J.A. (2018). The prescription drug problem we are missing: Risks associated with the misuse of tranquilizers and sedatives. J. Adolesc. Health.

[48] Erdsiek F., Özcebe H., Üner S., Karadag Caman Ö., Brand H., Czabanowska K., Gershuni O., Westerling R., Daryani A., Aksakal T. (2018). 2.5-O7 Rational drug use and migration: Awareness and attitudes towards antibiotic use among adults in Turkey and Turkish migrants in Sweden, the Netherlands and Germany. Eur. J. Public Health.

[49] Teli A., Kumar P., Singh A.D.T., Saini V. (2018). Drug use Evaluation Study in a Tertiary Care Corporate Hospital with special reference to use of Restricted Antibiotics in ICU Department. Glob. J. Pharm. Educ. Res..

